# Sodium Houttuyfonate Ameliorates *β*-amyloid_1-42_-Induced Memory Impairment and Neuroinflammation through Inhibiting the NLRP3/GSDMD Pathway in Alzheimer's Disease

**DOI:** 10.1155/2021/8817698

**Published:** 2021-05-31

**Authors:** Yuequan Zhao, YunPeng Tian, Tao Feng

**Affiliations:** ^1^The Second Ward of Neurology Department, Dongying People's Hospital, Dongying, Shandong 257091, China; ^2^Department of Pharmacy, Dongying People's Hospital, Dongying, Shandong 257091, China

## Abstract

**Objective:**

Our research is designed to explore the function of sodium houttuyfonate (SH) on Alzheimer's disease (AD) and its potential molecular mechanisms.

**Methods:**

In our study, the Morris water maze (MWM) test was used to assess the role of SH on spatial learning and memory deficiency in amyloid-*β* peptide (A*β*)_1-42_-induced AD mice. We explored the functions of SH on proinflammatory cytokines, neuron apoptosis, and damage *in vivo* and *in vitro* by using an enzyme-linked immunosorbent assay (ELISA), quantitative real-time polymerase chain reaction (qRT-PCR), flow cytometry, western blot, and Nissl staining. Moreover, the effect of SH on oxidative stress *in vivo* and *in vitro* was also detected. To explore the underlying molecular mechanisms of SH on AD, the expressions of proteins and mRNA involved in the NOD-like receptor pyrin domain containing-3/gasdermin D (NLRP3/GSDMD) pathway were determined using western blot, immunofluorescence staining, and qRT-PCR.

**Results:**

Our data demonstrated that SH ameliorated spatial learning and memory deficiency in A*β*_1-42_-induced AD mice. Moreover, SH significantly improved hippocampal neuron damage and inhibited oxidative stress, neuroinflammation, and neuron apoptosis in A*β*_1-42_-induced AD mice and PC12 cells. The results also revealed that SH protected A*β*_1-42_-induced AD through inhibiting the NLRP3/GSDMD pathway.

**Conclusion:**

The present study demonstrated that SH could ameliorate A*β*_1-42_-induced memory impairment neuroinflammation and pyroptosis through inhibiting the NLRP3/GSDMD pathway in AD, suggesting that SH may be a potential candidate for AD treatment.

## 1. Introduction

Alzheimer's disease (AD) is the most common neurodegenerative disease, which is mainly characterized by progressive cognitive impairment and irreversible memory loss [1, 2]. Recently, numerous studies have shown that the excessive production and deposition of amyloid-*β* peptides (A*β*) as senile plaques can initiate the process of neurodegeneration in AD [3, 4]. There is a newly diagnosed AD case every 3 sec all over the world [5]. However, current treatment and drug could not effectively terminate or reverse the progression of AD. Therefore, it is urgent to search new therapeutic methods and medicines for the treatment of AD.

*Houttuynia cordata* is a commonly used traditional Chinese herb and possesses a variety of pharmacological activities, including anti-inflammatory activity, antivirus activity, and antibacterial activity [6]. Decanoyl acetaldehyde (houttuynin) is one of the main extracts from *Houttuynia cordata*. At present, sodium houttuyfonate (C_12_H_23_O_5_SNa, SH) was synthesized by decanoyl acetaldehyde and sodium bisulfite as a result of the instability of decanoyl acetaldehyde [7]. A study by Huh et al. [8] has reported that *Houttuynia cordata* could significantly improve cognitive deficits in AD. However, the effect of SH on AD has not been reported.

NOD-like receptors (NLRs) are a kind of cytoplasmic sensors or receptors that play an important role in the process of neuroinflammation [9]. NOD-like receptor pyrin domain containing-3 (NLRP3) inflammasome, a member of the NLR family, is a multiprotein complex consisting of the core protein NLRP3 and regulatory molecules procaspase-1 and apoptosis-associated speck-like protein (ASC). The activated NLRP3 inflammasome causes the cleavage of procaspase-1 into active caspase-1, which can induce the secretion of proinflammatory cytokines [10, 11] and cleaves gasdermin D (GSDMD) into N-terminal pore-forming domain (GSDMD-N) and the C-terminal repressor domain (GSDMD-C) [12]. Subsequently, GSDMD-N interacts with the cell membrane for forming pores. Meanwhile, proinflammatory cytokines flow out through the pores and then cause inflammatory responses causing pyroptosis [12, 13]. Pyroptosis is an inflammatory form of programmed cell death that is associated with inflammasome activation [14]. Recently, increasing evidence has confirmed that the inhibition of pyroptosis can significantly prevent the progression of AD [15]. In addition, neuroinflammation is reported to be an important factor in the pathological procession of neurodegenerative disease, especially AD [16]. In this study, we detected the function of SH on AD and further investigated whether the mechanism is related to the NLRP3/GSDMD pathway.

## 2. Materials and Methods

### 2.1. Preparation of A*β* Oligomer

A soluble A*β* oligomer was prepared as described by Xiang et al. [17]. A*β*_1-42_ was dissolved in sterile H_2_O at a concentration of 1 mM, evaporated with high-purity N_2_ blowing at 37°C for 30 min, and then incubated for 48 h at room temperature to form the A*β* oligomer. Subsequently, the stock solution of A*β*_1-42_ was diluted in sterile phosphate-buffered saline (PBS) to the desired concentrations immediately before use.

### 2.2. Animals

Male ICR mice (6 weeks old, 25-30 g) were obtained from Vital River Experimental Technology Company (Beijing, China). All mice had free access to water and food and were maintained under a temperature of 22 ± 1°C, 60% humidity, and a 12 h light/dark cycle (lights on from 7:00 a.m. to 7:00 p.m.). Before the experiments, the animals were allowed to acclimate for seven days. The experimental protocol of our study was performed in accordance with the Guide for the Care and Use of Laboratory Animals and approved by the ethics committee of our hospital.

### 2.3. A*β*_1-42_ Injection and Drug Administration

Fifty mice were randomly divided into five treatment groups with ten animals in each group: Control group, A*β*_1-42_ group, A*β*_1-42_+SH (25 mg/kg) group, A*β*_1-42_+SH (50 mg/kg) group, and A*β*_1-42_+SH (100 mg/kg) group. Each mouse was anesthetized with intraperitoneal injection of pentobarbital sodium (40 mg/kg). Then, 3 *μ*l A*β*_1-42_ (10 ng/*μ*l) was stereotaxically injected into the hippocampus CA1 subregion in which the coordinates were as follows: -2.3 mm anterior/posterior, ±1.8 mm medial/lateral, and -1.75 mm dorsal/ventral from the bregma. In addition, the control group mice received the same volume of saline. From the second day after surgery, the mice in the A*β*_1-42_+SH (25 mg/kg), A*β*_1-42_+SH (50 mg/kg), and A*β*_1-42_+SH (100 mg/kg) groups were administered by oral administration with corresponding doses once a day, while mice in the Control and A*β*_1-42_ groups were administered with the same volume of saline for 28 days.

### 2.4. Morris Water Maze (MWM) Test

The MWM test was used to evaluate the spatial learning and memory abilities of the mice. Simply, following the given SH for 28 consecutive days, each animal was subjected to finding the submerged platform through a daily acquisition test (4 trials per day). And the maximum trial length was 120 s. If a mouse did not locate the platform within 120 s, it was then guided to the platform and placed on the platform for 30 s. In this case, its escape latency was still marked as 120 s. This procedure lasted for 6 days, with two training sessions each day and a 15 min interval between the sessions. On the sixth day, the animals were subjected to a probe trial session in which the platform was removed from the pool and mice were allowed to search for it for 120 s. The escape latency, the time spent in the target quadrant, and the frequency of mice crossing the virtual platform were recorded and analyzed by using a video tracking system.

### 2.5. Nissl Staining

The brain tissues of the mice were removed and postfixed in 4% formaldehyde solution at room temperature for 24 h. Subsequently, the brain tissues were dehydrated in graded concentrations of ethanol and then embedded in paraffin and transversely cut into 5 *μ*m thick sections. Then, the tissue sections were treated with conventional Nissl staining and visualized with an optical microscope (magnification, ×400).

### 2.6. Cell Culture and the Establishment of AD Model In Vitro

The rat pheochromocytoma (PC12) cell line was supplied by the Type Culture Collection of the Chinese Academy of Sciences (Shanghai, China). The cells were cultured in a RPMI-1640 medium (Sigma, USA) containing 10% fetal bovine serum (FBS; Gibco, USA) and 1% antibiotics (HyClone, USA) and maintained at 37°C in a humidified incubator containing 5% CO_2_.

For AD cell model *in vitro*, PC12 cells (1 × 10^4^ cells/well) were planted into 96-well plates and cultured for 24 h. The PC12 cells were pretreated with SH (15, 30, 60, 120, and 240 *μ*g/ml) or an inhibitor of NLRP3 inflammasome (CY-09, 10 *μ*M) for 2 h and then treated with A*β*_1-42_ (5 *μ*M) for 24 h at 37°C.

### 2.7. Cell Viability

The 3-(4,5-dimethylthiazol-2-yl)-2,5-diphenyltetrazolium bromide (MTT) assay was used to assess the cell viability. PC12 cells (1 × 10^5^ cells/well) were planted into 96-well plates and cultured overnight. After treatment of SH or A*β*_1-42_, 20 *μ*l of MTT solution (5 mg/ml, Beyotime, Nanjing, China) was added to each well and incubated at 37°C for about 4 h. After removal of the MTT solution, PC12 cells were treated with 200 *μ*l dimethyl sulfoxide (Beyotime) to dissolve the formazan crystals. Finally, the absorbance at 570 nm was recorded by using a microplate reader (Bio-Rad, USA).

### 2.8. Measurement of Reactive Oxygen Species (ROS), Malondialdehyde (MDA), and Superoxide Dismutase (SOD) Levels

To measure intracellular ROS level, PC12 cells (2 × 10^5^ cells/well) were planted into 6-well plates and cultured for 24 h. Following treatment of SH or A*β*_1-42_, PC12 cells were incubated with 10 nM fluorescent probe of 2′,7′-dichlorofluorescein diacetate (DCFH-DA, Sigma, USA) for 30 min at 37°C in the dark. The fluorescence signal was observed under a fluorescence microscope. In addition, the levels of SOD and MDA in both PC12 cells and brain tissues were measured by using the corresponding SOD and MDA detection kits (Beyotime) according to the manufacturer's instructions.

### 2.9. Flow Cytometry

The apoptosis of PC12 cells was assessed by using an Annexin V-fluorescein isothiocyanate/propidium iodide (FITC/PI) apoptosis detection kit (KeyGen Biotech, Shanghai, China) according to the instructions of the manufacturer. In brief, after treatment of SH or A*β*_1-42_, PC12 cells were harvested and resuspended in a binding buffer. Afterwards, the cells were stained by Annexin V-FITC and PI in the dark for 20 min. Finally, the cell apoptosis was detected by flow cytometer.

### 2.10. Immunofluorescence Staining

PC12 cells were fixed in 4% paraformaldehyde solution at room temperature for 20 min and then blocked with 5% bovine serum albumin (BSA) in PBS for 30 minutes at room temperature. After that, the slices were incubated with rabbit anti-GSDMD antibody (1 : 100, #20770-1-AP, Proteintech, USA) and rabbit anti-NLRP3 antibody (1 : 200, #ab214185, Abcam, USA) overnight at 4°C. Following washed with PBS, the slices were incubated with Cy3-labeled goat anti-rabbit secondary antibody (1 : 500, Beyotime) for 1 h at 37°C in the dark. The cells were stained with 4′,6-diamidino-2-phenylindole (DAPI, Sigma, USA) for 10 min at room temperature. The fluorescence images were randomly scanned with a confocal laser scanning microscope (Nikon, Tokyo, Japan) by a single investigator who was blind to the sample identity.

### 2.11. Enzyme-Linked Immunosorbent Assay (ELISA)

After treatment of SH or A*β*_1-42_, the levels of tumor necrosis factor *α* (TNF-*α*), interleukin-1*β* (IL-1*β*), interleukin-6 (IL-6), and interleukin-18 (IL-18) in both PC12 cells and brain tissues were detected by using their corresponding ELISA kits (R&D, USA) according to the instructions of the manufacturer.

### 2.12. Quantitative Real-Time Polymerase Chain Reaction (qRT-PCR)

To extract the total RNA from PC12 cells, a TRIzol reagent (Invitrogen, USA) was used following the manufacturer's manual. Total RNA was reverse-transcribed into cDNA by using a GoScript™ Reverse Transcription System (Promega, USA). Then, qRT-PCR was performed using a GoTaq one-step real-time PCR kit (Promega). The primer sequences were supplied by the Shanghai Rui Jingsheng Biological Engineering Co., Ltd. and listed as follows: NLRP3 (sense): 5′-GCTGGTCTTGAATTCCTCA-3′ and (antisense): 5′-GGCACACGGATGAGTCTTT-3′; ASC (sense): 5′-CTTGTCAGGGGATGAACTCAAAA-3′ and (antisense): 5′-GCCATACGACTCCAGATAGTAGC-3′; caspase-1 (sense): 5′-CCGTGGAGAGAAACAAGGAGT-3′ and (antisense): 5′-CCCCTGACAGGATGTCTCCA-3′; GSDMD (sense): 5′-GTGCCTCCACAACTTCCTGA-3′ and (antisense): 5′-GTCTCCACCTCTGCCCGTAG-3′; GAPDH (sense): 5′-CCACTCACGGCAAATTCAAC-3′ and (antisense): 5′-CTCCACGACATACTCAGCAC-3′.

### 2.13. Western Blot Analysis

Total protein was extracted from PC12 cells and brain tissues by using a RIPA lysis buffer (Beyotime). Protein quantification was performed using the Bradford method. The proteins were separated by 10% sodium dodecyl sulfate-polyacrylamide gel electrophoresis (SDS-PAGE) and transferred onto nitrocellulose membranes. After being blocked for 2 h in 5% nonfat dry milk, the membranes were incubated with the specific primary antibodies (Bcl-2, 1 : 1000, #12789-1-AP; Bax, 1 : 500, #15422-1-AP; caspase-3, 1 : 1000, #19677-1-AP; GSDMD, 1 : 500, #20770-1-AP; *β*-actin, 1 : 1000, #14395-1-AP; Proteintech, USA; NLRP3, 1 : 1000, #ab214185; ASC, 1 : 1000, #ab155970; caspase-1, 1 : 1000, #ab179515; Abcam, USA) overnight at 4°C. After being washed three times, the membranes were incubated with the horseradish peroxidase-conjugated secondary antibody (1 : 5000, Proteintech) for 2 hours at room temperature. Finally, the target proteins on the nitrocellulose membrane were visualized using the enhanced chemiluminescence (ECL) kit and captured using a Bio-Rad BioImaging System.

### 2.14. Statistical Analysis

All data were expressed as mean ± standard deviation and were analyzed by the SPSS22.0 software and GraphPad Prism 8.0 Software. One-way ANOVA combined with Turkey's multiple comparison tests was used to compare data. *P* values less than 0.05 were considered statistically significant.

## 3. Results

### 3.1. SH Ameliorates Spatial Learning and Memory Deficiency in A*β*_1-42_-Induced AD Mice

**Figure 1:**
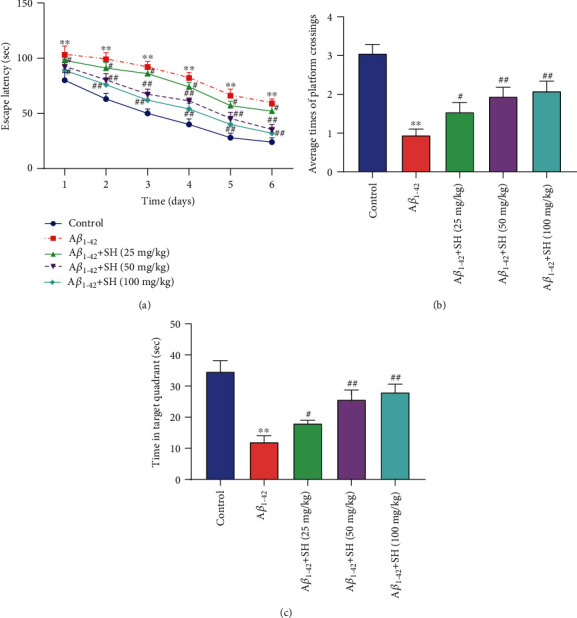
SH ameliorated spatial learning and memory deficiency in A*β*_1-42_-induced AD mice. (a) The escape latency of mice in the navigation test of the MWM test. (b) Time spent in the target quadrant during the probe trial. (c) The frequency of passing through the platform during the probe trial. ^∗∗^*P* < 0.01, vs. the Control group; ^#^*P* < 0.05, ^##^*P* < 0.01, vs. the A*β*_1-42_ group.

The MWM test was used to evaluate the function of SH on the spatial learning and memory in mice. As shown in Figure 1(a), the escape latencies of the Control group, A*β*_1-42_ group, A*β*_1-42_+SH (25 mg/kg) group, A*β*_1-42_+SH (50 mg/kg) group, and A*β*_1-42_+SH (100 mg/kg) group were progressively decreased in successive trials. When compared with the Control group, the escape latency of the A*β*_1-42_ group was significantly increased at 6 days (*P* < 0.01). SH (25, 50, and 100 mg/kg) dramatically decreased the escape latency at 6 days in a dose-dependent manner (*P* < 0.01). In addition, the results of Figures 1(b) and 1(c) showed that mouse swimming time in the target quadrant and the frequency of platform crossing in the A*β*_1-42_ group were significantly decreased in comparison with those in the Control group (*P* < 0.01). The mice treated with SH (25, 50, and 100 mg/kg) spent more time in the target quadrant and increased the frequency of platform crossing relative to the mice in the A*β*_1-42_ group. In the subsequently experiments, SH (50 mg/kg) was used. All the results suggested that SH could ameliorate spatial learning and memory deficiency in A*β*_1-42_-induced AD mice.

### 3.2. SH Attenuates A*β*_1-42_-Induced Hippocampal Alterations and Neuroinflammation in Mice

**Figure 2:**
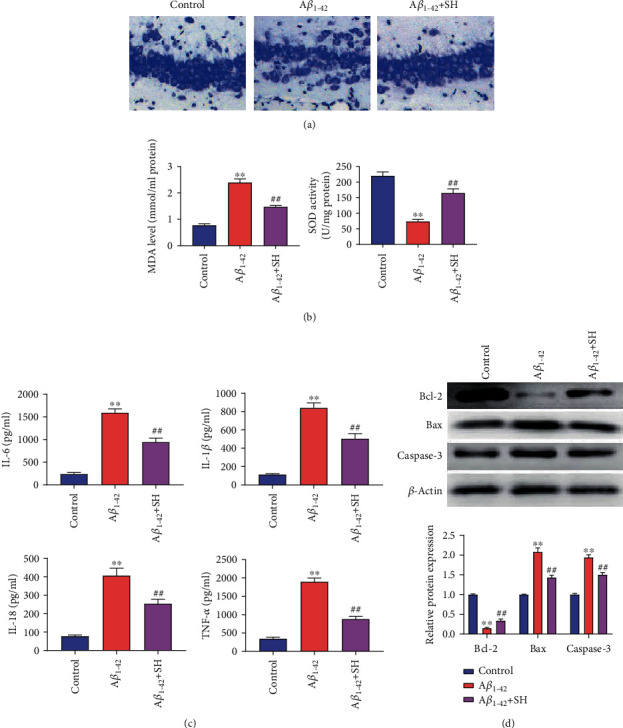
SH attenuated A*β*_1-42_-induced hippocampal alterations, oxidative stress, neuroinflammation, and neuron apoptosis in mice: (a) Nissl staining; (b) the content of MDA and SOD in each group; (c) the levels of IL-6, IL-1*β*, IL-18, and TNF-*α* in brain tissues were detected by ELISA; (d) the expressions of Bcl-2, Bax, and caspase-3 in brain tissues were detected by western blot. ^∗∗^*P* < 0.01, vs. the Control group; ^##^*P* < 0.01, vs. the A*β*_1-42_ group.

We firstly assessed the effects of SH (50 mg/kg) on hippocampus neuron survival by using Nissl staining (Figure 2(a)). The shrinkage and loss of the neurons were found in mice of the A*β*_1-42_ group relative to the Control group. When compared with the A*β*_1-42_ group, the treatment of SH (50 mg/kg) dramatically restored the impairment of neuronal loss and shrinkage. As Figure 2(b) showed, the MDA level in A*β*_1-42_ group brain tissues was significantly increased compared with that in the Control group (*P* < 0.01), while the SOD level was markedly decreased (*P* < 0.01). Treatment with SH (50 mg/kg) dramatically attenuated the role of A*β*_1-42_ on MDA and SOD (*P* < 0.01). The ELISA assay (Figure 2(c)) was further used to detect the levels of TNF-*α*, IL-1*β*, IL-6, and IL-18 in brain tissues. The results showed that the levels of IL-6, IL-1*β*, IL-18, and TNF-*α* in the A*β*_1-42_ group were higher than those in the Control group (*P* < 0.01). On the contrary, treating SH (50 mg/kg) significantly reduced the levels of IL-6, IL-1*β*, IL-18, and TNF-*α* compared with the A*β*_1-42_ group (*P* < 0.01). In addition, the results of the western blot (Figure 2(d)) revealed that the expressions of caspase-3 and Bax in A*β*_1-42_ group brain tissues were significantly increased compared with those in the Control group (*P* < 0.01), while the Bcl-2 expression was markedly decreased (*P* < 0.01). Treatment with SH (50 mg/kg) dramatically attenuated the effect of A*β*_1-42_ on the expressions of Bcl-2, Bax, and caspase-3 (*P* < 0.01). All the results indicated that SH (50 mg/kg) could attenuate A*β*_1-42_-induced hippocampal alterations, oxidative stress, neuroinflammation, and neuronal apoptosis in mice.

### 3.3. SH Attenuates A*β*_1-42_-Induced Oxidative Stress and Cell Apoptosis in PC12 Cells

**Figure 3:**
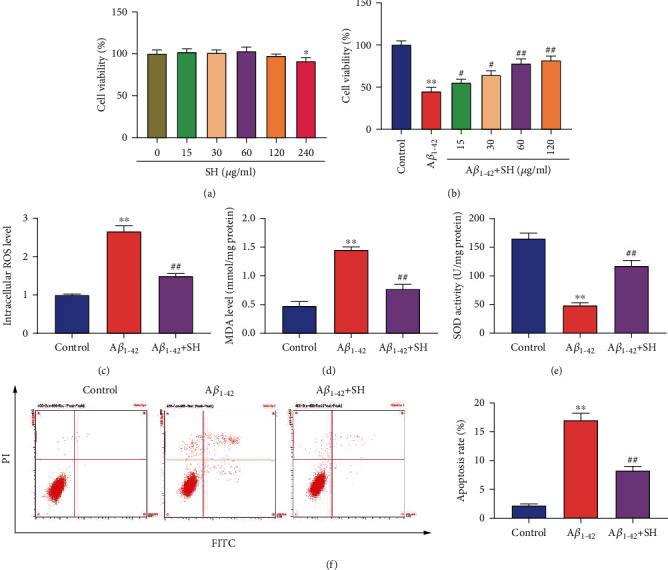
SH attenuated A*β*_1-42_-induced oxidative stress and cell apoptosis in PC12 cells. (a) Cell viability following treatment with SH at a range of concentrations was detected by MTT assay. (b) Cell viability following treatment with A*β*_1-42_ and SH at a range of concentrations was detected by MTT assay. (c) ROS level was detected with DCFH-DA and analyzed by fluorescence microscopy. (d) MDA level was determined with the MDA kit. (e) SOD level was determined with the SOD kit. (f) PC12 cells apoptosis was detected by flow cytometry. ^∗^*P* < 0.05, vs. SH (0 *μ*g/ml) group (a). ^∗∗^*P* < 0.01, vs. Control group; ^#^*P* < 0.05, ^##^*P* < 0.01, vs. A*β*_1-42_ group (b–f).

We explored the cytotoxic effect of SH on PC12 cell viability by using the MTT assay. The results showed that SH was not cytotoxic to PC12 cells in the concentration range of 0 *μ*g/ml to 120 *μ*g/ml (Figure 3(a)). In addition, the treatment with SH (15, 30, 60, and 120 *μ*g/ml) significantly inhibited the A*β*_1-42_-induced cell death in a dose-dependent manner (*P* < 0.05, *P* < 0.01) (Figure 3(b)). And there was no significant difference in the rescue effect of SH (60 *μ*g/ml) and SH (120 *μ*g/ml). Therefore, SH (60 *μ*g/ml) was chosen for the subsequent experiments. Then, we further investigated the effect of SH on A*β*_1-42_-induced oxidative stress and cell apoptosis in PC12 cells. As shown in Figures 3(c)–3(e), the levels of ROS and MDA in A*β*_1-42_ group PC12 cells were markedly elevated compared with those in the Control group (*P* < 0.01), but the SOD level was notably decreased (*P* < 0.01). Meanwhile, treating SH (60 *μ*g/ml) significantly attenuated the effect of A*β*_1-42_ on ROS, MDA, and SOD in PC12 cells (*P* < 0.01). Flow cytometry showed that A*β*_1-42_ significantly promoted PC12 cell apoptosis (*P* < 0.01) (Figure 3(f)). The treatment of SH (60 *μ*g/ml) dramatically reduced PC12 cell apoptosis when compared with the A*β*_1-42_ group (*P* < 0.01) (Figure 3(f)). All data demonstrated that SH (60 *μ*g/ml) could attenuate A*β*_1-42_-induced oxidative stress and cell apoptosis in PC12 cells.

### 3.4. SH Inhibits the Expressions of Inflammatory Factors in A*β*_1-42_-Induced PC12 Cells

**Figure 4:**
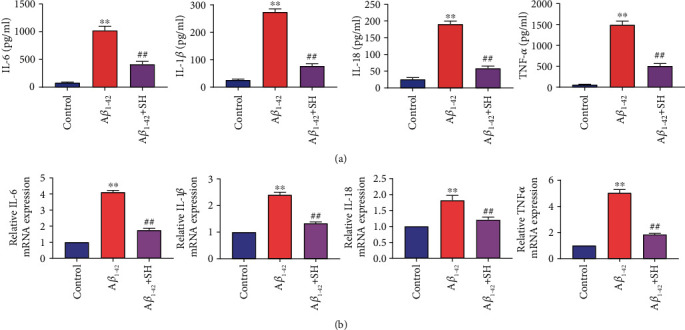
SH inhibited the expressions of inflammatory factors in A*β*_1-42_-induced PC12 cells. (a) The levels of IL-6, IL-1*β*, IL-18, and TNF-*α* were detected by ELISA. (b) The mRNA expressions of IL-6, IL-1*β*, IL-18, and TNF-*α* were detected by qRT-PCR. ^∗∗^*P* < 0.01, vs. the Control group; ^##^*P* < 0.01, vs. the A*β*_1-42_ group.

We investigated the effect of SH on the expressions of inflammatory factors in PC12 cells by using ELISA (Figure 4(a)) and qRT-PCR (Figure 4(b)). ELISA results showed that the levels of IL-6, IL-1*β*, IL-18, and TNF-*α* in the A*β*_1-42_ group were higher than those in the Control group (*P* < 0.01). Meanwhile, treating SH (60 *μ*g/ml) significantly decreased the levels of IL-6, IL-1*β*, IL-18, and TNF-*α* in comparison with the A*β*_1-42_ group (*P* < 0.01). Besides, qRT-PCR results also demonstrated that the mRNA expressions of IL-6, IL-1*β*, IL-18, and TNF-*α* were increased in the A*β*_1-42_ group compared with the Control group (*P* < 0.01). Treating SH significantly attenuated the effect of A*β*_1-42_ on the mRNA expressions of IL-6, IL-1*β*, IL-18, and TNF-*α* in PC12 cells (*P* < 0.01). The results above suggested that SH could inhibit the expressions of inflammatory factors in A*β*_1-42_-induced PC12 cells.

### 3.5. SH Protects A*β*_1-42_-Induced AD through Inhibiting the NLRP3/GSDMD Pathway in PC12 Cells

**Figure 5:**
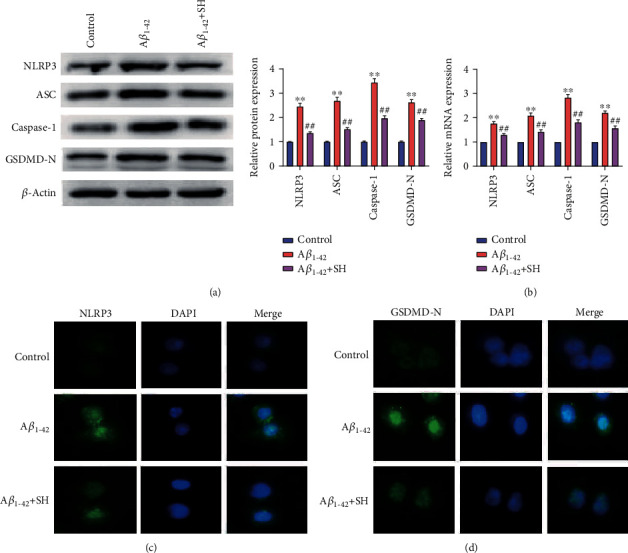
SH inhibited the NLRP3/GSDMD pathway in PC12 cells. (a) The protein expressions of NLRP3, ASC, caspase-1, and GSDMD-N were measured by western blot. (b) The mRNA expressions of NLRP3, ASC, caspase-1, and GSDMD-N were measured by qRT-PCR. (c) The expression of NLRP3 was determined by immunofluorescence staining. (d) The expression of GSDMD-N was determined by immunofluorescence staining. ^∗∗^*P* < 0.01, vs. the Control group; ^##^*P* < 0.01, vs. the A*β*_1-42_ group.

**Figure 6:**
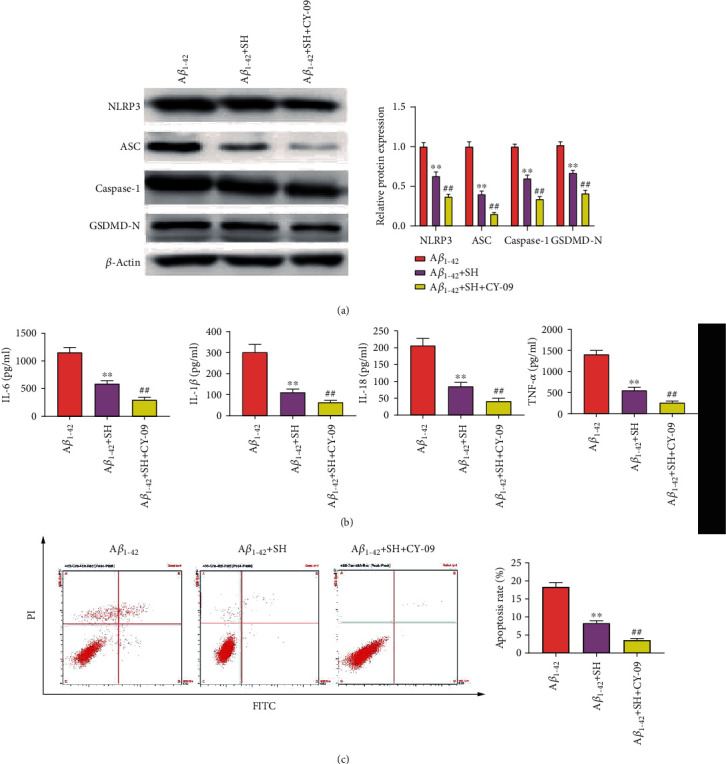
CY-09 enhanced the inhibitory effects of SH on proinflammatory cytokines and cell apoptosis in PC12 cells. (a) The protein expressions of NLRP3, ASC, caspase-1, and GSDMD-N were measured by western blot. (b) The levels of IL-6, IL-1*β*, IL-18, and TNF-*α* were detected by ELISA. (c) PC12 cell apoptosis was detected by flow cytometry. ^∗∗^*P* < 0.01, vs. the A*β*_1-42_ group; ^##^*P* < 0.01, vs. the A*β*_1-42_+SH group.

As shown in Figures 5(a) and 5(b), the protein and mRNA expressions of NLRP3, ASC, caspase-1, and GSDMD-N were significantly increased in the A*β*_1-42_ group compared with the Control group (*P* < 0.01). Meanwhile, treating SH (60 *μ*g/ml) dramatically decreased the protein and mRNA expressions of NLRP3, ASC, caspase-1, and GSDMD-N when compared with the A*β*_1-42_ group (*P* < 0.01). In addition, we evaluated the expression of NLRP3 and GSDMD-N in PC12 cells by immunofluorescence staining (Figures 5(c) and 5(d)). The results showed that the fluorescence intensities of NLRP3 and GSDMD-N were markedly higher in the A*β*_1-42_ group than the Control group, and these increases were notably attenuated by SH (60 *μ*g/ml). The data indicated that SH might protect A*β*_1-42_-induced AD through inhibiting the NLRP3/GSDMD pathway in PC12 cells. To further verify the hypothesis above, CY-09 was used. The results of the western blot showed that the expressions of NLRP3, ASC, caspase-1, and GSDMD-N were significantly decreased in the A*β*_1-42_+CY-09 group compared with the A*β*_1-42_+SH group (*P* < 0.01) (Figure 6(a)). As Figure 6(b) showed, the levels of IL-6, IL-1*β*, IL-18, and TNF-*α* in the A*β*_1-42_+CY-09 group were lower than those in the A*β*_1-42_+SH group (*P* < 0.01). In addition, flow cytometry also revealed that PC12 cell apoptosis was markedly decreased in the A*β*_1-42_+CY-09 group in comparison with the A*β*_1-42_+SH group (*P* < 0.01) (Figure 6(c)). All the results demonstrated that SH (60 *μ*g/ml) could protect A*β*_1-42_-induced AD through inhibiting the NLRP3/GSDMD pathway in PC12 cells.

## 4. Discussion

AD is a specific form of dementia in elderly people that affects more than 35 million individuals all over the world [18]. By 2050, the number of AD patients will increase to 135.46 million [19]. Although some progress has been made in the aspects of understanding the pathophysiology of AD, there are no effective treatments, and only a few medicines are currently available to patients. Thus, there is an urgent need to search innovative therapeutic strategies and effective drugs for the benefit of AD patients. In the present study, we confirmed that SH could ameliorate A*β*_1-42_-induced memory impairment, neuroinflammation, and pyroptosis through inhibiting the NLRP3/GSDMD pathway in AD.

### 4.1. Effect of SH on Cognitive Deficit and Memory Loss in AD Mice

The main pathological features of AD are progressive cognitive impairment and irreversible memory loss [20]. Huh et al. [8] have reported that *Houttuynia cordata* could significantly improve cognitive deficits of AD mice. Therefore, SH was selected in our study to investigate its effects on AD treatment. In the present study, MWM test results confirmed that SH significantly ameliorated spatial learning and memory deficiency in A*β*_1-42_-induced AD mice, suggesting that SH could improve cognitive deficits and memory loss in the progression of AD mice.

### 4.2. Effect of SH on A*β*_1-42_-Induced Neuroinflammation In Vivo and In Vitro

SH has been reported to have therapeutic effects on various diseases due to its anti-inflammatory activity and antibacterial activity [21–24]. Researchers have found that neuroinflammation plays a critical role in the progression of AD [25]. Singhal et al. have suggested that neuroinflammation is induced by the increased generation of inflammatory factors [26]. Demirci et al. have reported that the levels of proinflammatory cytokines (IL-6, IL-1*β*, IL-18, and TNF-*α*) in AD patients were higher than those in healthy volunteers [27]. In this study, we found that A*β*_1-42_ treatment significantly increased the levels of IL-6, IL-1*β*, IL-18, and TNF-*α* both *in vivo* and *in vitro*. SH significantly decreased the levels of IL-6, IL-1*β*, IL-18, and TNF-*α* in A*β*_1-42_-induced AD *in vivo* and *in vitro*. These data suggested that SH could prevent A*β*_1-42_-induced AD through inhibiting neuroinflammation.

### 4.3. Effect of SH on A*β*_1-42_-Induced Neuron Pyroptosis In Vivo and In Vitro

Galimberti and Scarpini [28] have reported that neuroinflammation is closely associated with neuronal apoptosis in AD. Neuronal apoptosis is identified to play an important role in learning and memory deficiency in AD [29]. In the present study, the results of the western blot showed that SH could increase Bcl-2 protein expression and inhibit protein expression of Bax and caspase-3 in A*β*_1-42_-induced AD *in vivo* and *in vitro*. Nissl staining results also demonstrated that SH could markedly attenuate hippocampal neural damage in A*β*_1-42_-induced AD mice. The data of flow cytometry showed that SH markedly inhibited A*β*_1-42_-induced apoptosis of PC12 cells. Besides, MTT results showed that SH significantly increased cell viability of A*β*_1-42_-induced PC12 cells. The above results indicated that SH could prevent A*β*_1-42_-induced AD through inhibiting apoptosis and increasing cell viability of neurons.

### 4.4. Effect of SH on A*β*_1-42_-Induced Oxidative Stress In Vivo and In Vitro

Oxidative stress induced by ROS overproduction is considered to contribute to the pathogenesis of AD [30]. Therefore, we focused on ROS and the origin of oxidative stress via determining the levels of oxidative stress markers (MDA and SOD). In our study, we found that A*β*_1-42_ treatment remarkably increased the MDA level and decreased the ROS level *in vivo* and *in vitro*. SH treatment significantly inhibited the generation of ROS and MDA and promoted the SOD level in A*β*_1-42_-induced AD *in vivo* and *in vitro*. The data confirmed that SH could prevent A*β*_1-42_-induced AD through inhibiting oxidative stress.

### 4.5. Effect of SH on the NLRP3/GSDMD Pathway in A*β*_1-42_-Induced PC12 Cells

Recently, more and more studies have indicated that inflammasomes are closely related to neurodegenerative diseases: activated NLRP3 was observed in AD [31]. It is reported that NLRP3 inflammasome plays important roles in A*β*-induced inflammation [32]. When NLRP3 inflammasome is activated, NLRP3 and ASC can cut procaspase-1 into active caspase-1 [33]. The active caspase-1 then induces a secondary cascade of events associated to the releases of proinflammatory cytokines such as IL-1*β* and IL-18 [10]. In addition, the active caspase-1 also cleaves GSDMD into GSDMD-N and GSDMD-C [12]. After that, inflammatory factors flow out from the cells through the pores caused by GSDMD-N, which further trigger an inflammatory response and cause pyroptosis [12]. Recently, increasing evidence has confirmed that the inhibition of pyroptosis can significantly prevent the progression of AD [15]. Our data showed that SH significantly decreased the expressions of NLRP3, ASC, caspase-1, and GSDMD-N in A*β*_1-42_-induced AD *in vitro*, suggesting that SH could inhibit the activation of the NLRP3/GSDMD pathway in AD. Then, to further verify whether SH could prevent AD through the NLRP3/GSDMD pathway, an inhibitor of NLRP3 inflammasome (CY-09) was used in our experiment. The results showed that the effects of SH on proinflammatory cytokines and cell apoptosis were enhanced by CY-09, indicating that SH could protect A*β*_1-42_-induced AD through inhibiting the NLRP3/GSDMD pathway.

The nosogenesis and progression of AD involve many factors. However, our present study was confined to exploring the mechanism of SH on the NLRP3/GSDMD pathway and oxidative stress. Whether SH can alleviate the symptoms of AD by other pathways remains to be further studied.

## 5. Conclusion

In summary, we demonstrated that SH could ameliorate A*β*_1-42_-induced memory impairment, neuroinflammation, and pyroptosis through inhibiting the NLRP3/GSDMD pathway in AD. The findings of our study reveal that SH could serve as a therapeutic approach for the treatment of AD. In the future, further study might be focused on other underlying mechanisms of SH on AD.

## Data Availability

The datasets used and analyzed during the current study are available from the corresponding author on reasonable request.
